# Supplementary material for: Investigating the influence of physiologically relevant hydrostatic pressure on CHO cell batch culture
Source: Sci Rep. 2021 Jan 8;11:162. doi: 10.1038/s41598-020-80576-8 (PMC7794228; doi:10.1038/s41598-020-80576-8)
Supplement: Supplementary file 1 — Supplementary Information 1. [file 41598_2020_80576_MOESM1_ESM.zip › S1/IgG/18Dec 4th round 5%CO2/blank.pdf]

===

Acq. Operator : SYSTEM Seq. Line : 3
Sample Operator : SYSTEM
Acq. Instrument : HPLC-0039 Location : 1
Injection Date : 12/18/2018 11:54:27 AM Inj : 1
Inj Volume : 100.000 µl
Sequence File : C:\Users\Public\Documents\ChemStation\1\Data\CHO_IgG 2018-12-18 11-44-16\CHO_IgG.S
Method : C:\Users\Public\Documents\ChemStation\1\Data\CHO_IgG 2018-12-18 11-44-16\CHO_IGG_LC.M (Sequence Method)
Last changed : 12/18/2018 11:44:20 AM by SYSTEM

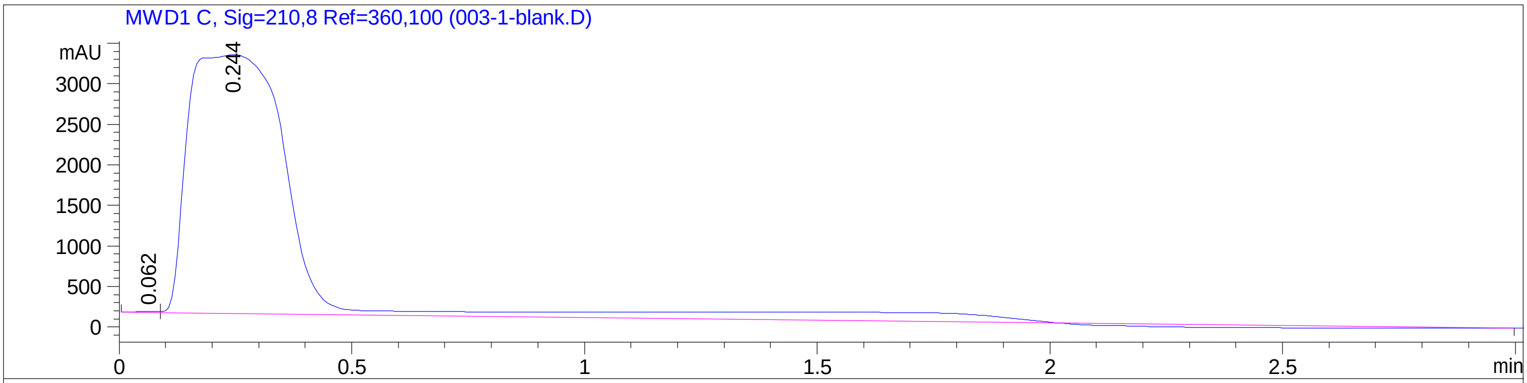

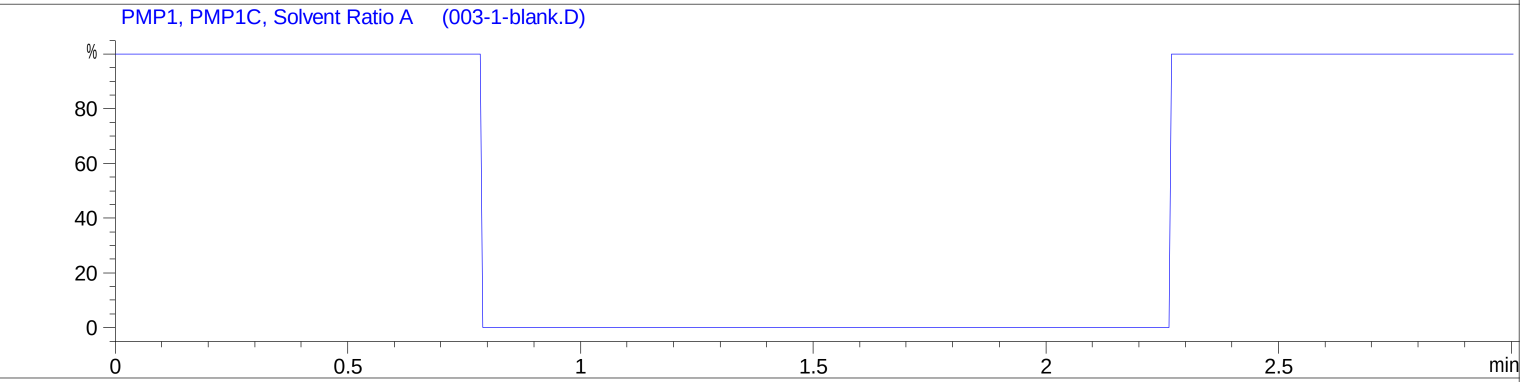

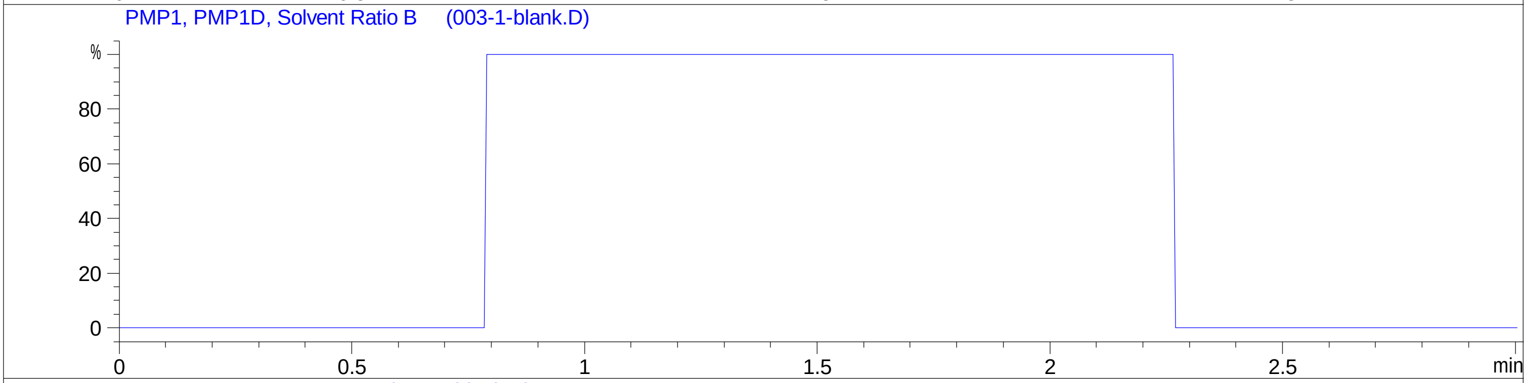

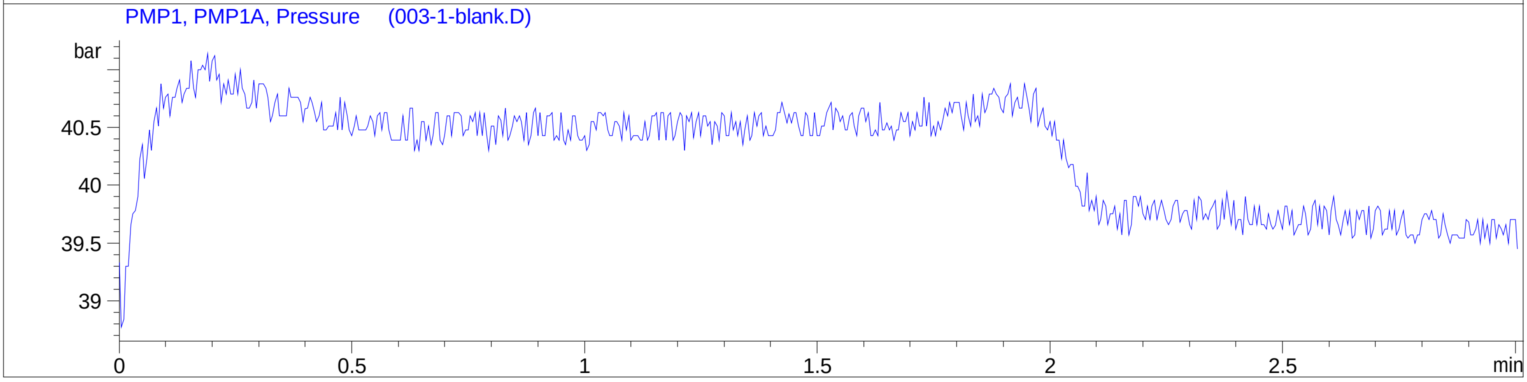

===

Area Percent Report

===

Sorted By : Signal
Multiplier : 1.0000
Dilution : 1.0000
Use Multiplier & Dilution Factor with ISTDs

Signal 1: MWD1 C, Sig=210,8 Ref=360,100

Peak #	RetTime [min]	Type	Width [min]	Area [mAU*s]	Height [mAU]	Area %
1	0.062	BV	0.0462	45.09202	15.37595	0.0909
2	0.244	VB	0.2231	4.95400e4	3190.68286	99.9091

Totals : 4.95851e4 3206.05881

===

*** End of Report ***
